# Verification and Validation of Advanced Control Systems for a Spinal Joint Wear Simulator

**DOI:** 10.3390/bioengineering11080779

**Published:** 2024-08-01

**Authors:** Kaushikk Ravender Iyer, David Keeling, Richard M. Hall

**Affiliations:** 1 Key Engineering Solutions Limited, Nexus Discovery Way, Leeds LS2 3AA, UK; d.g.keeling@key-es.co.uk; 2 School of Mechanical Engineering, University of Leeds, Woodhouse, Leeds LS2 9JT, UK; 3 College of Engineering and Physical Sciences, University of Birmingham, Edgbaston, Birmingham B15 2TT, UK; r.m.hall@bham.ac.uk

**Keywords:** Fuzzy-PI controller, SIMO fuzzy logic control system, Leeds spinal wear simulator, hardware-in-the-loop (HiL) simulation, activities of daily living (ADLs)

## Abstract

Wear simulation aims to assess wear rates and their dependence on factors like load, kinematics, temperature, and implant orientation. Despite its significance, there is a notable gap in research concerning advancements in simulator control systems and the testing of clinically relevant waveforms. This study addresses this gap by focusing on enhancing the conventional proportional–integral–derivative (PID) controller used in joint simulators through the development of a fuzzy logic-based controller. Leveraging a single-input multiple-output (SIMO) fuzzy logic control system, this study aimed to improve displacement control, augmenting the traditional proportional–integral (PI) tuning approach. The implementation and evaluation of a novel Fuzzy-PI control algorithm were conducted on the Leeds spine wear simulator. This study also included the testing of dailyliving (DL) profiles, particularly from the hip joint, to broaden the scope of simulation scenarios. While both the conventional PI controller and the Fuzzy-PI controller met ISO tolerance criteria for the spine flexion–extension (FE) profile at 1 Hz, the Fuzzy-PI controller demonstrated superior performance at higher frequencies and with DL profiles due to its real-time adaptive tuning capability. The Fuzzy-PI controller represents a significant advancement in joint wear simulation, offering improved control functionalities and more accurate emulation of real-world physiological dynamics.

## 1. Introduction

Joint wear simulators have been developed to provide insight into the wear performance of total joint replacements and preclinically evaluate implant designs and bearing materials [1,2,3,4,5,6]. Longer implantation periods and younger, heavier, and more active patients contribute to increased tribological demand on artificial joints [7,8]. Historically, the primary cause for the failure of most hip replacements has been wear [9,10,11,12,13]. Patient-specific factors like age, gender, weight, activity level, and individual kinematic patterns have been found to influence wear [14,15,16]. To ensure the tribological performance of joint arthroplasties, they undergo standard testing procedures before being introduced to the market. However, current regulatory preclinical testing standards, such as ISO 14242-1 and ISO 18192-1 [17,18], utilize simplified and stylized waveforms to define standardized loading conditions. These conditions do not accurately represent the loads and motion experienced by different patient groups in real-life activities [19].

The use of mechanical simulators is considered the pre-eminent method for realistically predicting how combinations of biomaterials and implant geometry will perform tribologically after total joint replacement [20,21]. Compared to the more simplistic pin-on-disk machines, simulators aim to replicate the actual tribological conditions experienced by the joint, which is derived from a more sophisticated design and electro-mechanical control [22,23]. The objective of wear simulation is to determine the wear performance of the implant or bearing combination and analyze its dependence on multiple factors, including load, kinematics, temperature, and the orientation of the sliding components within the implant [24,25]. However, another aspect that is lacking in the existing literature is advancements in the control system of the simulator and testing of clinically relevant waveforms pertaining to patients’ real-life activities [26,27]. To ensure that these simulators accurately reflect real-world situations, it is crucial to simulate more challenging scenarios and incorporate a wider range of daily activities, particularly for younger and more active patients [28]. The existing literature on joint simulators predominantly highlights advancements in control system development for in-vitro biomechanical testing of the spine, with relatively less emphasis on in vitro wear testing. The purpose of in vitro biomechanical testing is to examine the three-dimensional motion of the spine in response to loads. This is normally conducted on a cadaver spine by using either multiple spinal segments or a single functional spinal unit (FSU) [29,30,31,32,33,34,35,36]. 

There is a noticeable gap in the current literature concerning the preclinical evaluation of prostheses via in-vitro wear testing on joint simulators, specifically regarding the incorporation of daily living (DL) motion and load cycles for the spine, knee, and hip. These DL profiles typically stem from gait lab investigations involving a significant patient cohort participating in activities like walking, sitting, standing, stair ascent, and descent [25,37]. One factor contributing to this gap is the constraints that early joint simulators face due to limited degrees of freedom in motion and load. However, modern joint simulators, particularly the electro-mechanical ones discussed in [38] for the knee and hip joints from the University of Leeds, such as the one utilized in this study for the spine, are programmable and demonstrate improved adaptability in handling complex motion and loading parameters. Another reason for the absence of DL profile testing is the limited requirement in preclinical testing of joint prostheses to adhere solely to ISO standards, which encompass simplified gait cycles rather than the diverse real-life scenarios encountered by individuals [27]. The main rationale for integrating DL profiles into the testing protocol is that studies conducted by [39,40] reveal that the wear patterns observed on retrieved implants cannot be reproduced using existing standards. 

Therefore, this study addresses this gap by introducing a novel control algorithm—Fuzzy-PI—and its development and testing tailored to the Leeds spinal wear simulator. The algorithm’s effectiveness was evaluated by subjecting both ISO and DL profiles to the tolerances outlined in ISO 18192-1 for the cervical spine [17]. This paper focuses on developing and conducting proof-of-concept trials of an advanced control system for a joint simulator used in tribological testing of spinal prostheses. Two control algorithms, namely proportional–integral (PI) and Fuzzy-PI, were developed and tested. Utilizing the novel HiL environment [41] offered a robust platform to test both controllers rigorously across various ISO-compliant and daily living profiles of the spine, hip, and knee. The Leeds spine simulator was utilized as a testing platform to validate both fuzzy-PI and PI control algorithms based on its robustness and flexibility to test simple sinusoid profiles of the spine that are ISO compliant [17], as well as profiles representing activities of daily living (ADLs).

This paper aims to evaluate and compare the effectiveness of the conventional PI controller with the enhanced Fuzzy-PI controller within the context of a joint simulator. The objective is to confirm the performance of these controllers by aligning their outcomes with the tolerances outlined in the ISO-18192-1 standards for the cervical spine. The results present the findings from the HiL simulation and real-time performance assessments of both PI and Fuzzy-PI controllers on the Leeds spinal wear simulator. It includes comparative analyses of controller responses to ISO and daily living profiles, illustrating the superior performance of the Fuzzy-PI controller. The Section 4 interprets the results, emphasizing the advantages of the Fuzzy-PI control system over conventional PI controllers. It also discusses the novelty of using fuzzy logic-based PID controllers in joint simulators and the potential for broader applications.

## 2. Materials and Methods

In this paper, a novel intelligent controller employing a Fuzzy-PI algorithm was applied to the Leeds SimSol spine simulator (Simulator Solutions Ltd., Manchester, UK). The primary purpose was to assess its effectiveness in conducting wear tests on tribological implants. The Fuzzy-PI control system underwent an initial phase of development and thorough testing using a universal Hardware-in-the-Loop (HiL) setup [41]. This HiL environment was specifically designed to evaluate potential control algorithms prior to integrating them into the operational framework of the Leeds spine simulator [41,42]. Before testing Fuzzy-PI and PI controllers on the spine wear simulator, the following preceding developmental milestones were achieved:

- Designed a novel HiL test bench: A Hardware-in-the-Loop (HiL) test bench was created to facilitate the development, rapid prototyping, and testing of prospective control algorithms against ISO standards [42].
- Developed and tested a PI control algorithm: Initially, a velocity-based PI control algorithm was developed and tested on the HiL test bench using speed profiles derived from the displacement curves of the ISO and hip ADL [42].
- Benchmarked with PI Controller: The PI controller was established as a benchmark to test and verify the minimum motion control requirements outlined by ISO 18192-1, which involves a simple sinusoidal flexion–extension (FE) profile at 1 Hz.
- Developed and tested a Fuzzy-PI control algorithm: An advanced PI control algorithm based on fuzzy logic, known as Fuzzy-PI, was developed on the HiL test bench to extend the testing capabilities by simulating ISO profiles at 2 Hz and ADL profiles of the hip, such as walking [41]. The performance of the Fuzzy-PI controller was compared to the PI controller using a Bode plot, and a frequency sweep was conducted for the ISO-FE profiles at 0.5 Hz, 1 Hz, 1.5 Hz, and 2 Hz [41].

### 2.1. Spine Wear Simulator

**Figure 1:**
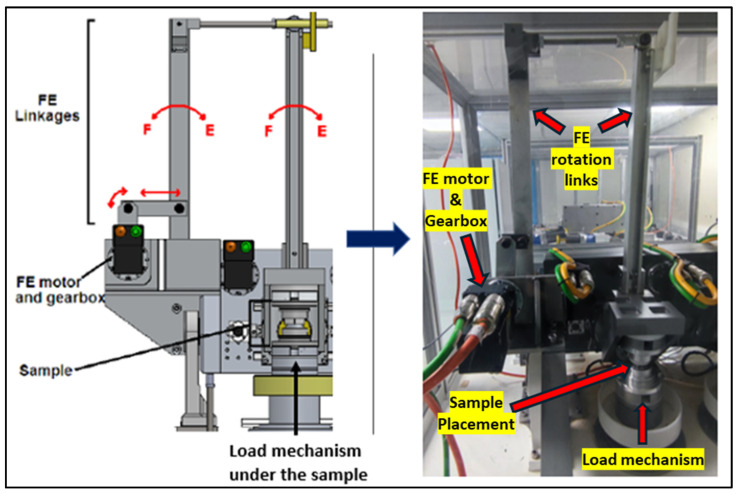
Schematic of FE linkages driven by the FE motor of the Leeds spinal wear simulator [23].

Figure 1 presents a segment of the spinal wear simulator situated at the University of Leeds. This apparatus employs electro-mechanical actuators to regulate both kinetic and kinematic inputs. Precise motion control is facilitated by harmonic-drive motors, which have previously demonstrated the required performance in this context. The simulator is capable of achieving six degrees of freedom, with five degrees effectively operational.

Two velocity-based control algorithms, proportional–integral (PI) and Fuzzy-PI, were developed and tested. The innovative HiL environment provided a robust platform to rigorously develop and test both controllers across ISO-compliant and daily living profiles for the spine, hip, and knee [41]. The Leeds spine simulator validated both Fuzzy-PI and PI control algorithms, demonstrating its robustness and flexibility in testing simple sinusoidal profiles from ISO-18192-1 for the spine and profiles representing activities of daily living (ADLs). The test profiles were selected based on their waveform configuration, frequency component bandwidth, and relevance to human ADLs.

During control prototyping on the HiL system [41,42], the PI controller struggled to overcome phase lag at higher frequencies (e.g., 2 Hz) as specified in the ISO 18192-1 standards, requiring frequent manual adjustments to K_p_ and K_i_ gains. To address these issues, the Fuzzy-PI controller was developed.

### 2.2. Fuzzy-PI Controller Design

This paper introduces an innovative intelligent control algorithm that combines fuzzy logic (FL) with a proportional–integral (PI) controller, specifically designed for tribological testing on joint simulators. The derivative (D) term was excluded due to its sensitivity to external disturbances, which caused instability with its aggressive response [43]. 

The proposed Fuzzy-PI controller seeks to synergistically complement the simplicity and robustness of PI, whereby the fuzzy logic (FL) controller acts as a supervisor to the PI controller and optimally selects gains (K_p_ and K_i_) in real-time as the test unfolds. 

The non-linear characteristics of a fuzzy controller make it more effective in capturing and representing complex behaviors compared to a classical linear PID controller [44]. To the best of the authors’ knowledge, no literature exists on the application of the Fuzzy-PI control algorithm in the field of wear testing of joint prostheses using a joint simulator at the time of this research. However, numerous studies have shown that the Fuzzy-PI controller surpasses the conventional PI controller in various applications, demonstrating enhanced performance and adaptability in dynamic environments [45,46,47,48]. The Fuzzy-PI controller developed for this study utilizes a set of predefined K_p_ and K_i_ gains that are dynamically adjusted in real-time, based on the changing shape and frequency of the demand profile.

**Figure 2:**
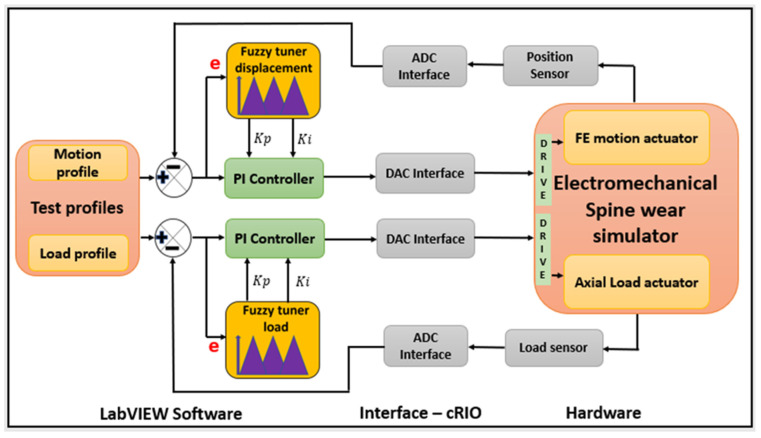
Control architecture for the Leeds electro-mechanical spinal wear simulator.

Figure 2 provides a schematic to describe the advanced Fuzzy-PI control methodology developed.

The two controllers were comparatively tested against a selection of profiles. Each test ran for 100 cycles. However, some hip ADL profiles such as climbing up and down a staircase, stumbling, and tibial internal–external rotation of the knee ran only for 20–40 cycles because of its extreme complexity in shape and the frequency components therein. These profiles were trialed to test the robustness of both PI and Fuzzy-PI controllers and the capability of the spine simulator to simulate profiles of other joints, like the hip and knee. 

The profile frequency was adjusted across four values: 0.5 Hz, 1 Hz, 1.5 Hz, and 2 Hz, with 2 Hz being the maximum allowable test frequency according to ISO standards. The controller’s performance was assessed using metrics outlined in ISO 18192-1 (accuracy of ±0.5° at the maxima and minima of the motion and ±2% of the full cycle time for phasing) [17].

### 2.3. Fuzzy Logic Membership Function Design

Triangular membership functions (MFs) are among the most frequently used MFs in practical applications [49]. These functions are constructed with straight lines, offering a straightforward design [50]. The choice of MF shape often depends on the specific problem at hand. A comprehensive review of various studies reveals that triangular MFs are favored for their simplicity [46,51,52,53,54,55,56]. When comparing different MFs, such as Gaussian and triangular, the latter consistently performs well, often outperforming others. Zhao and Bose [57] evaluated the system’s response with different MFs and concluded that triangular MFs are the most effective.

**Figure 3:**
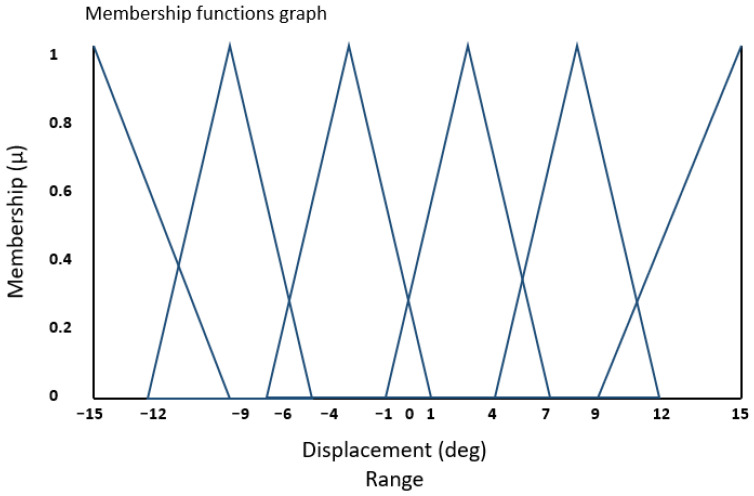
Input fuzzy membership function for displacement error (e).

Figure 3 displays the fuzzy triangular membership function for the displacement error (e). The horizontal X-axis represents the universe of discourse spanning from −15 to +15. This range encompasses the numerical values defined by the system designer, aligning with the controller’s specifications and requirements. The X-axis was carefully selected to cover the minimum and maximum values of all profiles tested in this study.

**Figure 4:**
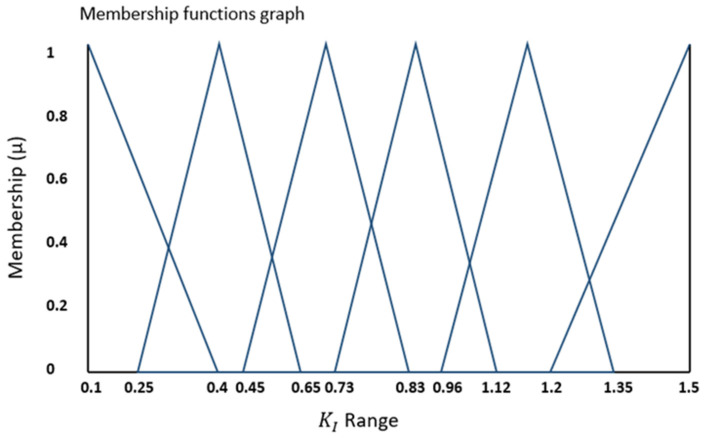
Output fuzzy membership function for $K_{i}$ gain.

**Figure 5:**
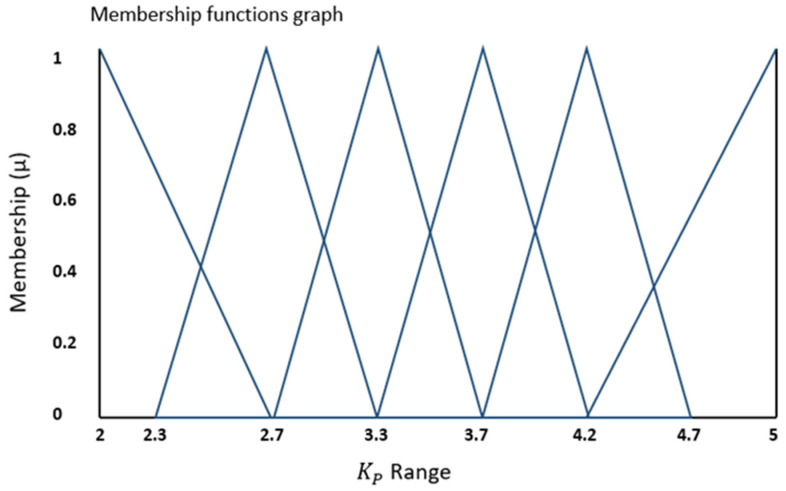
Output fuzzy membership function for $K_{p}$ gain.

Additionally, Figure 4 and Figure 5 depict the complete range of integral ($K_{i}$) and proportional ($K_{p}$) gains for the error values falling within the range outlined in Figure 3. In these figures, the vertical Y-axis represents the degree of membership (µ), which takes values within the range of {0, 1}.

**Figure 6:**
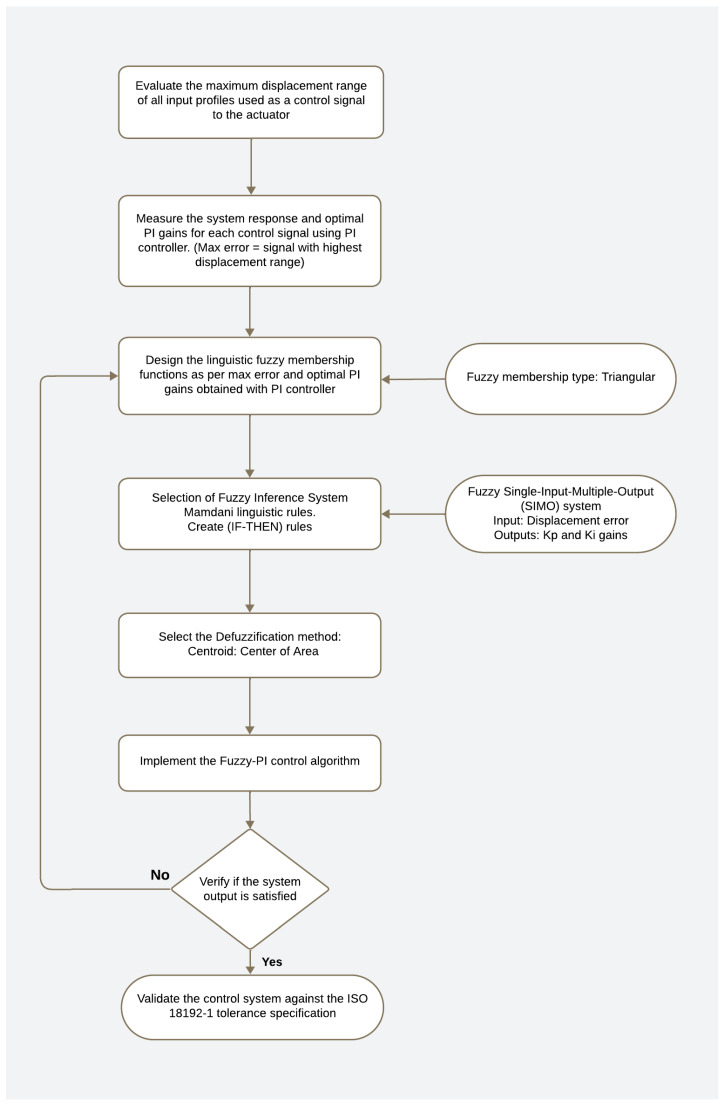
Fuzzy logic control development process.

Table 1 showcases the assessment of fuzzy rules derived from the fuzzy membership functions, while Figure 6 outlines the process steps for developing the fuzzy logic controller, which supervises the PI controller. 

## 3. Results

**Figure 7:**
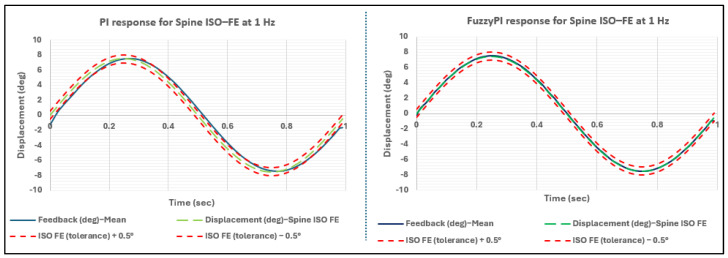
Proportional–integral (PI) vs. fuzzy–proportional–integral (Fuzzy-PI) response of spine ISO flexion–extension (FE) at 1 Hz.

To validate the real-time performance of both PI and Fuzzy-PI controllers on the Leeds spinal wear simulator, an initial assessment was conducted utilizing a standardized flexion–extension (FE) profile delineated in ISO 18192-1 [17]. Subsequently, a walking profile of the hip obtained from the gait lab measurements at Leeds Teaching Hospital Trust underwent testing, owing to its intricate shape, frequency components, and direct relevance to human activities of daily living (ADLs). The findings presented illustrate the mean response observed over 100 simulated cycles at frequencies of 1 Hz and 2 Hz. Figure 7 exhibits the control responses of both controllers against the target FE displacement profile prescribed in ISO 18192-1. As illustrated in Table 2, the Fuzzy-PI controller demonstrated superior performance over the industry standard PI controller in terms of phase lag as per the tolerances stipulated in the ISO 18192-1 standards. Nevertheless, the PI controller adhered to the ISO tolerance limit for both amplitude and phase, proving its efficacy despite being outperformed by the Fuzzy-PI controller in terms of phase lag. 

**Figure 8:**
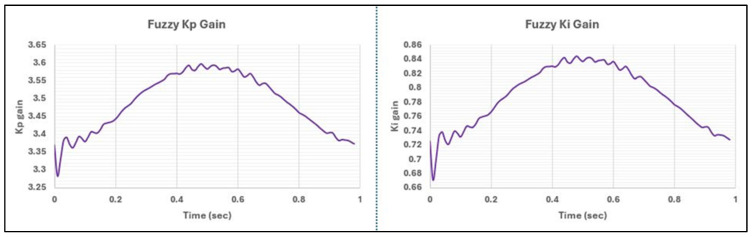
Fuzzy gains for spine ISO-FE profile at 1 Hz.

The performance difference in phase between the two controllers can be attributed to the tuning process of the PI controller on the Leeds spine simulator. Specifically, the PI controller was fine-tuned for the 1 Hz profile, with parameters set to K_p_ = 3.5 and K_i_ = 0.73, through iterative testing across a range of profiles, including the daily living hip profiles. In contrast, the Fuzzy-PI controller, instead of being fixed to a singular value, followed a range of K_i_ and K_p_ gains, as depicted in Figure 4 and Figure 5 as Fuzzy Triangular membership functions. Figure 8 indicates the real-time auto-tuning of fuzzy gains to optimally align with the demand ISO FE profile.

**Figure 9:**
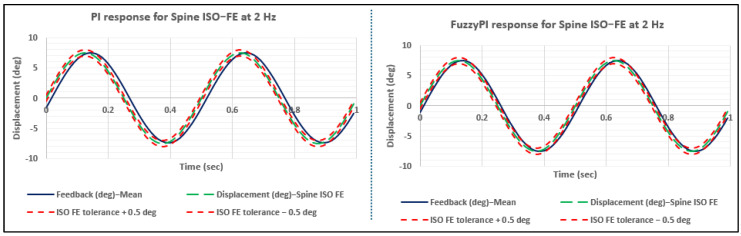
Proportional–integral (PI) vs. fuzzy–proportional–integral (Fuzzy-PI) response of spine ISO flexion–extension (FE) at 2 Hz.

**Figure 10:**
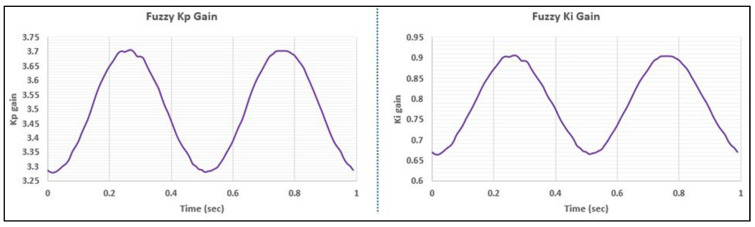
Fuzzy gains for Spine ISO-FE profile at 2 Hz.

Figure 9 illustrates the controllers’ responses to the spine ISO FE profile at a frequency of 2 Hz. As indicated in Table 2, for the 2 Hz scenario, the Fuzzy-PI controller consistently outperformed the PI controller in terms of phase lag. The Fuzzy-PI controller stayed closer to the ISO tolerance limits, exceeding them by only 0.2%, compared to the PI controller’s 1.6%. Moreover, Figure 10 depicts the dynamic auto-tuning of fuzzy gains replicating the sinusoidal characteristics of the original signal. These dynamic adjustments allow the system to effectively respond to varying conditions, maintaining stability and precision. The specified membership functions and rules facilitate the controller’s ability to adapt the gains dynamically, aligning with the system’s requirements at any moment. 

**Figure 11:**
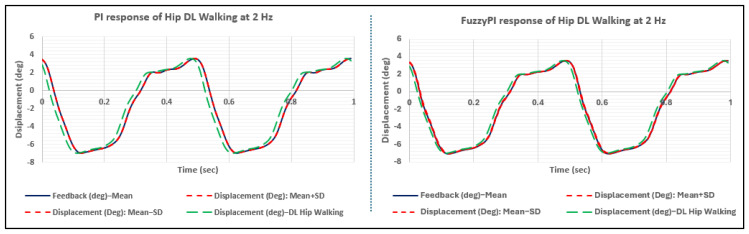
Proportional–integral (PI) vs. fuzzy–proportional–integral (Fuzzy-PI) response for hip daily living walking profile at 2 Hz.

**Figure 12:**
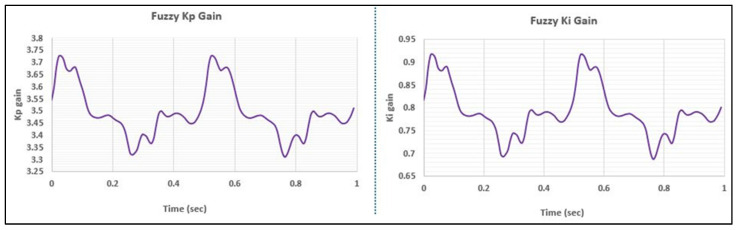
Fuzzy gains for hip daily living walking profile at 2 Hz.

Figure 11 presents the controllers’ responses to the real-world (daily living) walking profile of the hip, tested at a frequency of 2 Hz. Both controllers exhibited impressive performance; this marked a significant milestone, as such complex, non-sinusoidal daily living profiles for a different joint had not been previously tested on the Leeds spine simulator. Notably, even when subjected to the daily living profile, the Fuzzy-PI controller outperformed the PI controller in terms of phase lag, which is attributable to its dynamically adjusting gain values, as illustrated in Figure 12. 

Due to the absence of international standards for setting tolerance limits for daily living profiles, the ISO 18192-1 tolerance criteria for the spine were used for phase comparison. In this context, the Fuzzy-PI controller successfully met the tolerance requirements, while the PI controller exceeded 2%, thus failing to conform to the prescribed ISO limits detailed in Table 3.

## 4. Discussion

The HiL setup, as described in previous research [41,42], was custom-built to develop and evaluate potential controllers for the Leeds spine simulator. It offered comprehensive insights into the performance of two distinct control algorithms. Notably, there is no existing literature on using a fuzzy logic-based PID controller in a joint simulator for the wear testing of joint prostheses. This study is therefore the first to develop and implement a simple fuzzy logic controller based on a single-input multiple-output (SIMO) system, which acts as a supervisor to intelligently optimize the gains of the PI controller.

The results presented earlier in this section showed that the Fuzzy-PI control system met the tolerance specifications outlined in the ISO 18192-1 standards for the cervical spine, even when tested with the hip ADL profile at 2 Hz. This achievement is particularly notable as there is currently no literature documenting the testing of a hip ADL displacement profile at 2 Hz. Moreover, the findings in Section 3 underscore the intra-cycle performance advantages of Fuzzy-PI, owing to its intelligent gain optimization. This feature enables the control system to adhere to ISO profiles and adapt to adverse dynamic loading profiles across various cycle frequencies.

A significant advantage of the Fuzzy-PI control system over conventional industry-standard PI control systems is its user-friendly operation. Once developed with carefully selected membership function ranges, Fuzzy-PI eliminates the need for the manual retuning of controller gains, thus simplifying a system’s usage when compared to PI alone, which requires tuning for each variation in motion and load profiles. The results also illustrate how Fuzzy-PI optimizes gains in real-time, enhancing control throughout a cycle. Furthermore, there is potential for medium- and long-term performance benefits, whereby the dynamic intra-cycle tuning of Fuzzy-PI could mitigate changes in the contact surface or mechanical system such as wear, friction, and hydration to ensure that motion and load profiles do not deteriorate.

This research is a pioneering effort that investigates the application of a novel control system, Fuzzy-PI, in a spine wear simulator. The objective is to evaluate profiles that go beyond the basic sinusoidal ISO standards, incorporating more complex ADL profiles at frequencies higher than the standard 1 Hz. A significant finding of this study is the successful testing of an ADL profile at 2 Hz on a joint simulator, with the Fuzzy-PI controller generating a response that met ISO tolerances. 

The results of this thesis are crucial for both researchers and industries that employ joint simulators. They provide essential insights and practical solutions for tackling the complexities of real-life profiles, which frequently display non-sinusoidal behaviors. This study bridges a significant gap in existing research, establishing a foundational framework that enhances the development and evaluation of joint simulators. This advancement allows for more precise simulations and analyses of complex movements and activities that occur in real-world situations.

### Broader Applications and Achievements of Fuzzy Logic

The successful application of fuzzy logic in developing a novel control system for a spinal simulator in tribological applications has expanded its utility and demonstrated its benefits in other areas of medical engineering, including the identification and selection of optimal biomaterials for spinal implants [58]. The innovative Fuzzy-PI control algorithm can be utilized in various joint simulators designed for tribological testing, such as friction and wear assessment. Additionally, it holds promise for biomechanical testing, including the evaluation of real cadaveric functional spinal units, as detailed in [31,32,59]. Moreover, combining the FLC with other advanced intelligent control algorithms, such as artificial neural networks (ANNs), has proven effective in gait analysis for post-stroke patients [60].

## 5. Conclusions

In summary, this study has achieved substantial progress in enhancing control systems for spinal wear simulators. By overcoming the shortcomings of existing testing methodologies and introducing more intricate, real-world profiles, this research sets a foundation for future innovations in assessing and improving the preclinical evaluation of joint prostheses. The implementation of advanced control algorithms, such as Fuzzy-PI, in joint simulators, offers significant potential for boosting the accuracy, dependability, and overall efficiency of biomedical devices.

## 6. Future Work

This paper has demonstrated the use of a pioneering control system, Fuzzy-PI, alongside the conventional PI controller, for wear testing on a Leeds spinal wear simulator. Future work should focus on expanding the application of the Fuzzy-PI and PI controllers to include a broader range of activities of daily living (ADL) profiles, such as various spinal movements. Additionally, testing should be conducted over a longer duration, ideally spanning at least 1 million cycles, to thoroughly evaluate the controllers’ performance. It is also essential to integrate the biological environment surrounding the prosthesis, as specified in ISO standards, to accurately mimic real human body conditions. This comprehensive evaluation will facilitate rigorous testing and validation under diverse and demanding scenarios. Researchers and practitioners are encouraged to explore these avenues to further advance the understanding and application of these control systems in spinal wear simulation.

## Figures and Tables

**Table 1:** Fuzzy rules used to evaluate displacement error (e), with K_p_ and K_i_ expressed in linguistic form.

Sr No.	Fuzzy Rules
1	IF Displacement Error is “Large Neg”, THEN Kp is “Extreme”; ALSO, Ki is “Extreme”
2	IF Displacement Error is “Medium Neg”, THEN Kp is “Very Large”; ALSO, Ki is “Very Large”
3	IF Displacement Error is “Small Neg”, THEN Kp is “Large”; ALSO, Ki is “Large”
4	IF Displacement Error is “Small Pos”, THEN Kp is “Medium”; ALSO, Ki is “Medium”
5	IF Displacement Error is “Medium Pos”, THEN Kp is “Small”; ALSO, Ki is “Small”
6	IF Displacement Error is “Large Pos”, THEN Kp is “Very Small”; ALSO, Ki is “Very Small”

**Table 2:** Controller performance evaluation for Spine ISO FE against ISO 18192-1 tolerance specification.

Controller Performance vs. ISO 18192-1 Tolerance				RMS Error	
Frequency	Performance Metric	Fuzzy-PI	PI	Fuzzy-PI	PI
1 Hz	Amplitude (±0.5°)	±0.022°	±0.036°	0.07	0.62
	Phase (±2%)	−0.5° (0.13%)	−6.72° (−1.86%)		
2 Hz	Amplitude (±0.5°)	±0.1°	±0.2°	0.74	1.19
	Phase (±2%)	−7.97° (−2.2%)	−12.97° (−3.6%)		

**Table 3:** Controller performance evaluation for hip daily living walking conditions against the ISO 18192-1 tolerance specification.

Controller Performance vs. ISO 18192-1 Tolerance				RMS Error	
Frequency	Performance Metric	Fuzzy−PI	PI	Fuzzy-PI	PI
2 Hz	Amplitude (±0.5°)	±0.1°	±0.15°	0.49	0.81
	Phase (±2%)	6.2° (1.7%)	−10.3° (2.9%)		

## Data Availability

Data are available upon reasonable request to the corresponding author.
